# The impact of innate immunity and epigenetics in the pathogenesis of hidradenitis suppurativa

**DOI:** 10.3389/fimmu.2025.1593253

**Published:** 2025-05-19

**Authors:** Olivia M. Burke, Victoria R. Frerichs, Dario F. Garcia, Rivka C. Stone, Hadar Lev-Tov, Tali Czarnowicki, Robert W. Keane, Nkemcho Ojeh, Jelena Marjanovic, Irena Pastar, Marjana Tomic-Canic, Juan Pablo de Rivero Vaccari, Andrew P. Sawaya

**Affiliations:** ^1^ Dr. Phillip Frost Department of Dermatology and Cutaneous Surgery, Leonard M. Miller School of Medicine, University of Miami, Miami, FL, United States; ^2^ Wound Healing and Regenerative Medicine Research Program, Dr. Phillip Frost Department of Dermatology & Cutaneous Surgery, Miami, FL, United States; ^3^ Department of Physiology and Biophysics, Leonard M. Miller School of Medicine, University of Miami, Miami, FL, United States; ^4^ Department of Neurological Surgery and the Miami Project to Cure Paralysis, University of Miami Miller School of Medicine, Miami, FL, United States; ^5^ Department of Neurological Surgery, Leonard M. Miller School of Medicine, University of Miami, Miami, FL, United States; ^6^ Faculty of Medical Sciences, The University of the West Indies, Cave Hill, Cave Hill, Barbados

**Keywords:** hidradenitis suppurativa, innate immunity, epigenetics, inflammasomes, inflammatory skin disease

## Abstract

Hidradenitis Suppurativa (HS) is a chronic multifactorial inflammatory skin disease with a debilitating impact on quality of life. Here, we review the complex interplay of innate and adaptive immune dysregulation in HS pathogenesis, in the context of microbial dysbiosis, genetic predisposition, cellular dysfunction and epigenetic factors. Hyperactivation of the innate system triggered by follicular occlusion leads to a cascade of activated signaling pathways leading to persistent inflammation as the disease progresses. This immune hyperactivation is further complicated by microbiome dysbiosis, which is associated with dysregulation of inflammasomes and altered expression of host antimicrobial peptides. Keratinocytes, fibroblasts, macrophages, and neutrophils exhibit altered functions, and contribute to the inflammatory cascade and disease chronicity in HS. Epigenetic mechanisms including DNA methylation, histone modifications, and non-coding RNAs modulate immune responses and contribute to aberrant cytokine and chemokine expression that drive the persistent inflammatory state in HS pathogenesis. We highlight the need for future research to explore the concept of epigenetic memory in epidermal stem cells and inflammasome activation to gain a better understanding of these mechanisms and pave the way for development of future novel therapeutic targets and strategies to disrupt the persistent chronic inflammation cycle in this debilitating condition.

## Introduction

**Figure 1:**
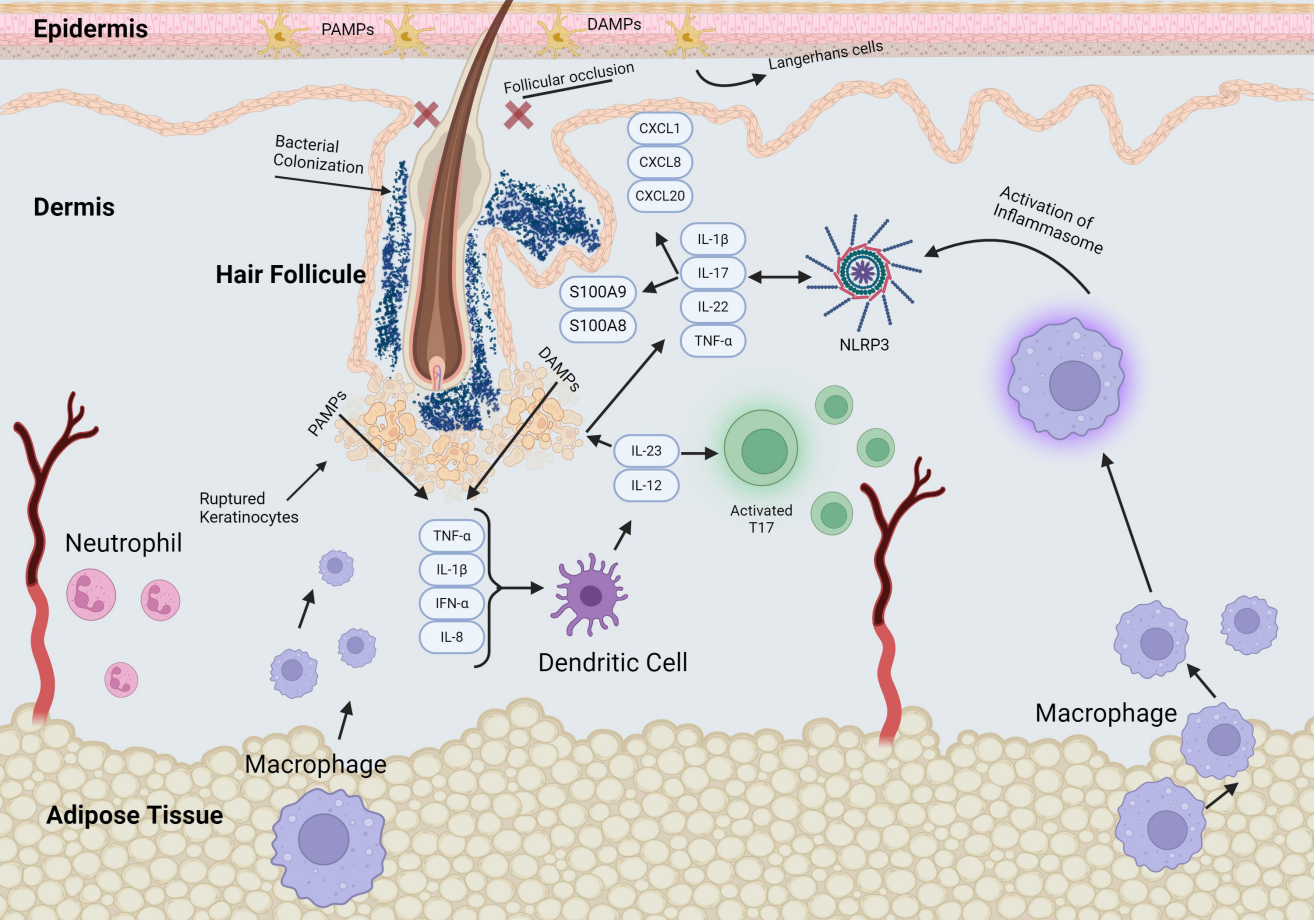
Schematic of signaling pathways in early stage of HS pathogenesis. The early events of HS pathogenesis include significant changes in perivascular and perifollicular immune cell infiltration, hyperkeratosis, and hyperplasia of the infundibular epithelium. Follicular occlusion and stasis promote bacterial proliferation and follicular dilation leading to rupture of the hair follicle, triggering release of pro-inflammatory mediators and recruitment of immune cells.

Hidradenitis Suppurativa (HS) is a chronic inflammatory disease that presents with painful lesions, including deep nodules, abscesses, tunnels, and fibrotic scars in intertriginous areas of the skin (1). HS has a global prevalence of up to 4%, with an estimated prevalence of 0.10% in the United States (2). This debilitating condition disproportionately affects African American and Hispanic women (3). However, the exact pathogenic mechanisms involved in the development and progression of HS are not fully understood. HS is believed to originate from the epithelium of hair follicles involving both terminal and vellus hairs. Follicular occlusion triggers a cascade in which ruptured follicles attract significant infiltrates to the affected area (**Figure 1**). When multiple follicles or cysts rupture, it leads to autoantigen exposure and inflammation (4). This environment becomes susceptible to bacterial colonization, ultimately resulting in abscess and tunnel formation (5).

**Figure 2:**
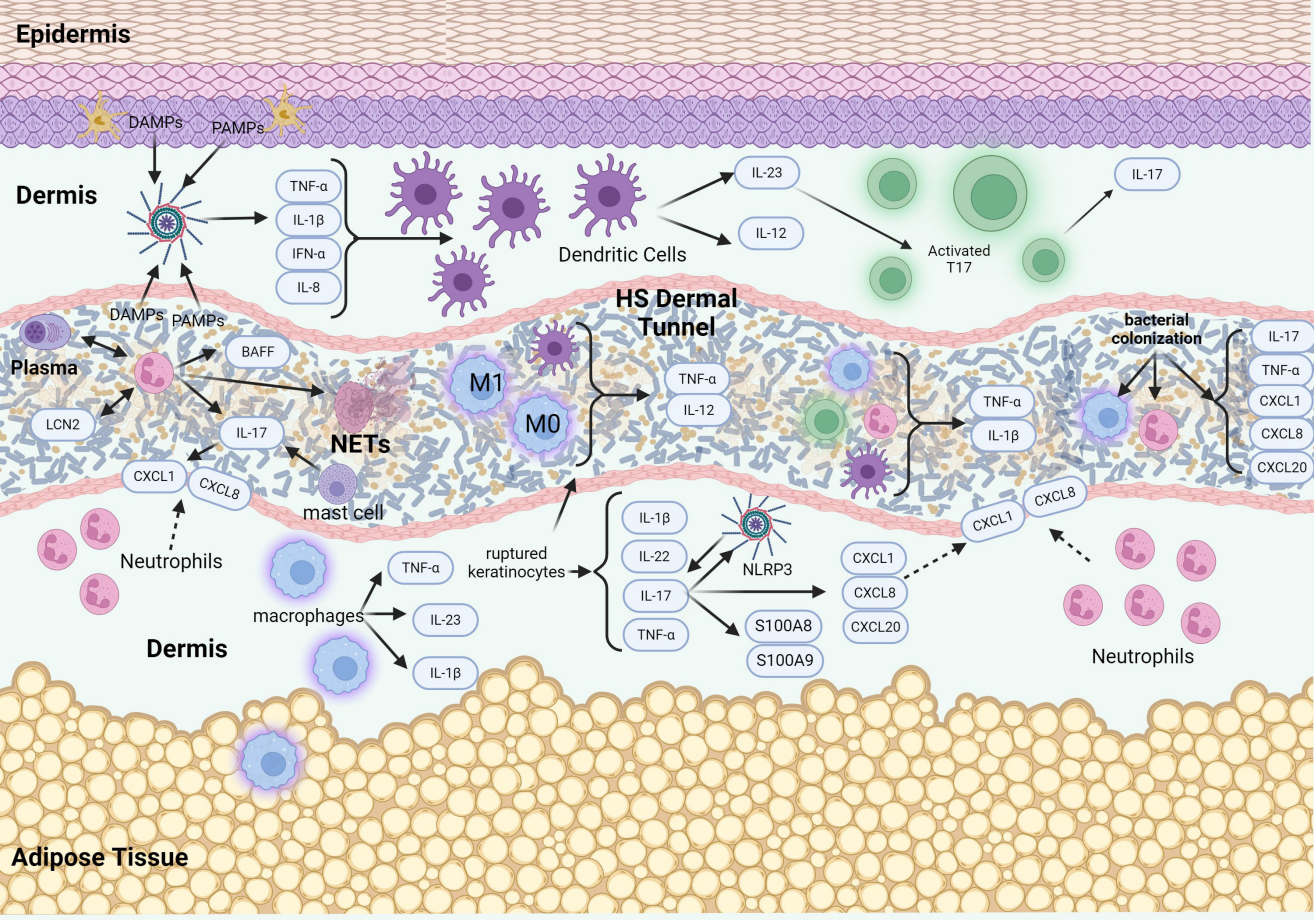
Schematic of signaling pathways in advanced stages of HS pathogenesis. The advanced stage of HS pathogenesis is characterized by an intense inflammatory response, leading to the destruction of hair follicles and the formation of abscesses and sinus tracts. Epithelialized tunnels are formed that harbor microbial colonization with pro-inflammatory mediators and immune cells that are contained within the lumen of the tunnels. The presence of tunnels further exacerbates the inflammatory response and contributes to disease chronicity and treatment resistance.

In the more advanced stages of HS, Hurley Stages 2 and 3, the condition is marked by extensive immune cell infiltration, including neutrophils, macrophages, dendritic cells, and T cells, which produce pro-inflammatory cytokines such as IL-1β and TNF (**Figure 2**). This inflammatory response results in recurrent abscesses accompanied by scarring and the formation of sinus tracts in Stage 2, and widespread involvement with interconnected sinus tracts and abscesses covering an entire region in Stage 3 (6).

Current treatment of HS typically involves a combination of medical therapies such as antibiotics, retinoids, and biologics, alongside lifestyle modifications and surgical interventions tailored to the severity of the disease. Biologics, particularly those targeting TNF-α and IL-17, have shown considerable promise. TNF-α inhibitors like adalimumab have demonstrated efficacy in reducing inflammation, decreasing the size of lesions, and improving clinical outcomes, especially in severe cases (7). Clinical trials such as the PIONEER I and II studies underscore adalimumab’s ability to achieve significant response rates compared to placebo (8). However, challenges such as recurrence post-treatment and potential tolerance development remain (9). In addition to anti-TNF therapies, recently anti-IL-17 agents like secukinumab and bimekizumab, were recently FDA-approved. (10–12). IL-17A is highly expressed in HS lesions, where it contributes to the inflammation and pathogenesis of the disease (13). Studies have shown that IL-17A is produced by various immune cells, including T cells, neutrophils, and mast cells, within the affected skin (13–15). Secukinumab showed significant improvement in clinical outcomes for moderate-to-severe HS in the SUNSHINE and SUNRISE phase 3 trials, with rapid efficacy observed by week 2 and sustained through week 52. Over 75% of patients who responded by week 16 maintained their clinical improvements, underscoring secukinumab’s potential as an effective treatment option (16). In addition, recent clinical trials demonstrated that dual inhibition of IL17A and IL17F with bimekizumab showed greater clinical improvement compared to placebo control groups (12, 17). These studies underscore the importance of IL17 signaling in HS pathogenesis and support the use of anti-IL17 biologics for the treatment of patients with moderate-to-severe HS.

In this review, we provide a comprehensive synthesis of emerging evidence on the interplay between innate immunity and epigenetic regulation in the pathogenesis of hidradenitis suppurativa. We explore how epigenetic mechanisms, such as DNA methylation, histone modifications, and non-coding RNAs, modulate immune cell activation, cytokine production, and barrier dysfunction, contributing to the chronic inflammatory state characteristic of HS. Special attention is given to the concept of epigenetic memory and its role in priming keratinocytes, fibroblasts, and immune cells toward persistent inflammatory responses. We also highlight the involvement of distinct immune cell subsets, including innate lymphoid cells, NK cells, and mast cells, as well as the contribution of fibroblast heterogeneity and inflammasome activation to disease progression. Environmental factors such as smoking, obesity, and diabetes are discussed as modulators of the epigenome, potentially reinforcing inflammatory circuits and impaired wound healing. This integrative framework identifies novel molecular targets and provides new insights into the mechanisms underlying HS chronicity and treatment resistance.

## Innate immune mechanisms involved in HS pathogenesis

Innate immune mechanisms are thought to play a significant role in the pathogenesis of HS and contribute to the complex interplay of inflammatory responses associated with this challenging and complex skin condition. Follicular occlusion leads to activation of the innate immune system. Toll-like receptors (TLRs) expressed by keratinocytes and Langerhans cells are activated by pathogen-associated molecular patterns (PAMPs) and damage-associated molecular patterns (DAMPs) (18). This activation prompts the release of pro-inflammatory cytokines, including tumor necrosis factor (TNF)-α, interferon (IFN)-α, interleukin (IL)-1β, IL-6 or IL-8 which activate dendritic cells. Activated dendritic cells secrete IL-12 and IL-23, with IL-23 playing a crucial role in T helper (Th)17 activation. HS is characterized by a pronounced Th1 and Th17 inflammatory profile (19). IL-23 facilitates keratinocyte proliferation and stimulates release of TNFα, IL-22, IL-1β, and IL-17. IL-17, in turn, stimulates keratinocytes to produce proinflammatory proteins (S100A8/A9) and activate the NLRP3 inflammasome, as well as the release of chemokines such as CXCL1, CXCL8, and CCL20 (20). Furthermore, IL-17 binding to IL-17RA receptor activates nuclear factor (NF)-κB leading to increased pro-inflammatory gene expression. In addition to these dendritic cell and T cell-mediated pathways, natural killer (NK) cells also contribute significantly to the innate immune dysregulation observed in HS. CD56^+^ NK cells co-localize with IL-32 in lesional skin and, in early disease, exhibit enhanced production of IFN-γ and granzyme B, both of which amplify local inflammation (21, 22). This heightened cytotoxic activity suggests that NK cells may play a prominent role in disease initiation. Notably, NK cell numbers appear to inversely correlate with time since disease onset, implying that their influence may diminish with chronicity (21). Beyond NK cells, another player in the innate immune landscape of HS is the innate lymphoid cell (ILC) population. ILCs are significantly expanded in HS and exhibit high CD2 expression, localizing to lesional skin regions involved in inflammation, fibrosis, and tunnel formation (23). These cells contribute to disease pathogenesis through cytotoxic activity and the secretion of pro-inflammatory cytokines. Notably, their activation can be attenuated by blocking the CD2:CD58 interaction, suggesting a potential therapeutic target (23). Further characterization of ILC subsets revealed total ILCs, particularly ILC2 and ILC3, are enriched in non-lesional compared to lesional HS skin (24). The presence of ILC3s, which produce IL-17 and IL-22 and are associated with Notch signaling and sebaceous gland regulation, supporting a role in early lesion formation and HS pathogenesis (24). While ILC1 levels were not elevated in non-lesional HS skin, they were the predominant ILC population in lesional skin, possibly contributing to ongoing inflammation (24). Additionally, anti-TNF treatment appeared to normalize ILC levels in blood, supporting the idea that ILCs migrate to the skin and may initiate or sustain inflammation in HS (24).

**Figure 3:**
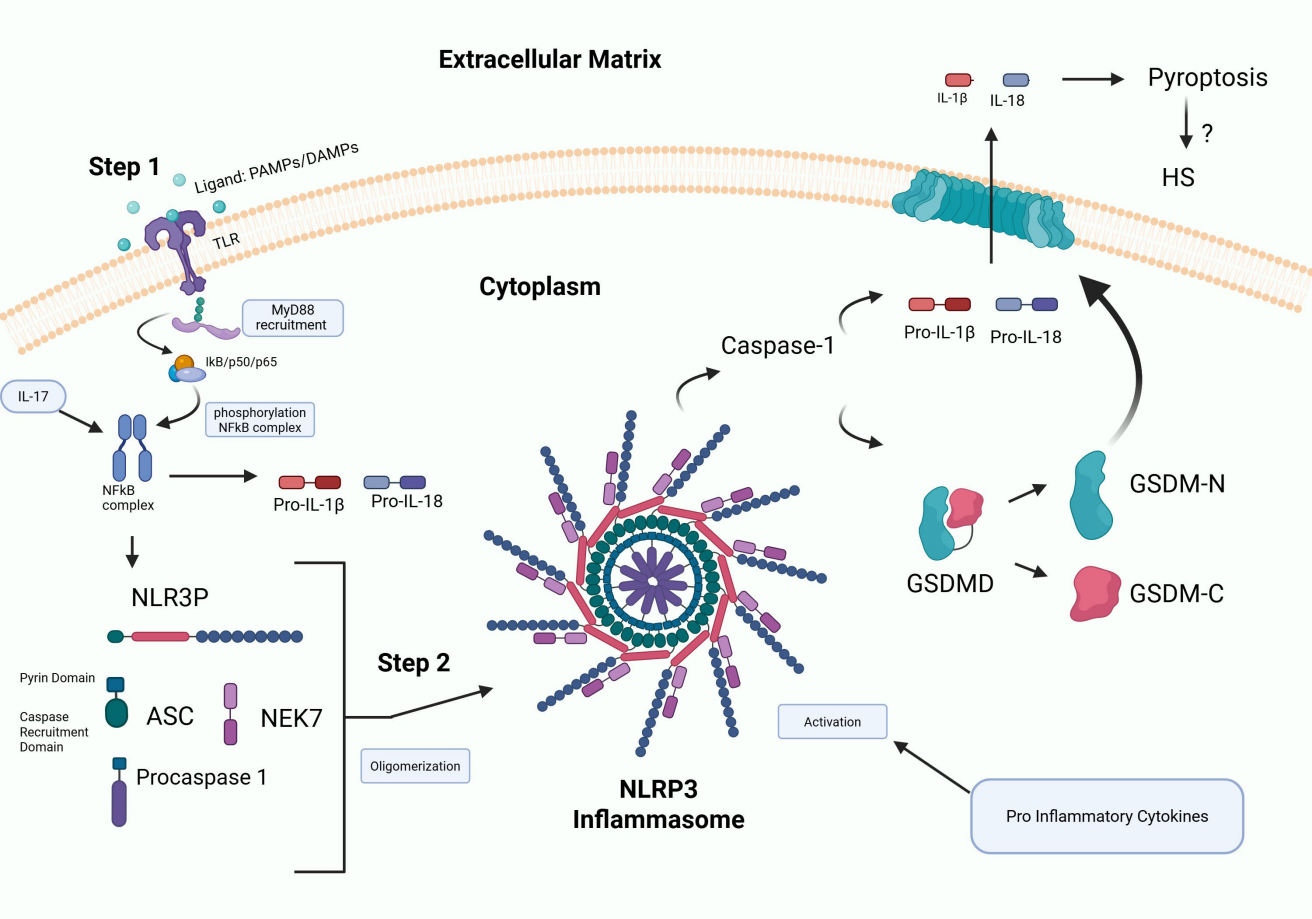
Schematic of inflammasome activation. Step 1: Priming involves binding of PAMPs and DAMPs to TLRs initiating NF-kB activation and upregulation of inflammasome signaling molecules. Step 2 involves NLRP3 inflammasome assembly that triggers cleavage of IL-1β and gasdermins by caspase-1 resulting in inflammation and pyroptosis, leading to cell rupture and release of pro-inflammatory mediators into the surrounding environment. NLRP3 has been shown to be induced in HS (28), supporting a role for fueling the highly inflammatory response.

Increasing evidence supports a role for the NLRP3 inflammasome in contributing to the pathogenesis of HS (25, 26). Inflammasomes are multi-protein complexes that are activated in response to various stimuli that include microbial ligands, danger-associated molecular patterns (DAMPs) and pathogen-associated molecular patterns (PAMPs), all of which are highly induced in HS (26). Activation of NLRP3 triggers cleavage of pro IL-1β (26). In addition, inflammasome activation promotes cleavage of gasdermin D (GSDMD) by caspase-1 triggering pyroptosis, an inflammatory form of cell death resulting in cell rupture and release of pro-inflammatory contents into the surrounding environment (27). NLRP3 has been shown to be induced in HS (28), supporting a role for inflammasomes in fueling the chronic inflammatory response in HS. This cascade results in the recruitment of inflammatory cytokines to both the follicular unit and the skin surrounding the lesions (**Figure 3**). Elevated levels of IL-1 family cytokines, particularly IL-1β and IL-36, have been observed in both lesional and non-lesional HS skin, suggesting that persistent inflammasome activation underlies systemic immune dysregulation in HS. This has led to the investigation of several IL-1–targeted therapies, including anakinra (IL-1 receptor antagonist), bermekimab (anti–IL-1α), canakinumab (anti–IL-1β), lutikizumab (dual IL-1α/β blockade), and IL-36 receptor inhibitors such as spesolimab and imsidolimab, as promising therapeutic strategies to mitigate chronic inflammation (29).

Antimicrobial peptides (AMPs) are key defensive molecules produced by epithelial and immune cells with direct antimicrobial properties (30). In HS, the expression of several AMPs is altered. One such AMP is Cathelicidin (LL-37), derived from hCAP18 encoded by the Cathelicidin antimicrobial peptide (CAMP) gene (31). LL-37 is induced in follicular keratinocytes and neutrophils in HS, contributing to inflammation and potentially fostering bacterial resistance (32). Furthermore, LL-37 promotes Th1/Th17 cell maturation and upregulates aforementioned pro-inflammatory cytokines like TNF-α and IL-17 (33). Human β-defensin (hBD)-2 levels are increased in HS, promoting inflammation and keratinocyte growth, whereas hBD-1 is decreased, possibly leading to microbial imbalance (34, 35). Furthermore, HS is associated with microbial dysbiosis, particularly in skin folds (36). This imbalance between beneficial and harmful bacteria exacerbates inflammation and promotes biofilm formation. Moreover, pathogens commonly found in HS lesions include *Staphylococcus aureus* and anaerobic bacteria such as *Porphyromonas* and *Prevotella* species, and biofilms allow bacteria to evade the innate immune response, and chronic biofilm formation in tunnels drive chronicity in this condition (37).

Unlike inducible peptides such as human cathelicidins and beta-defensins, which mainly act in response to injury and inflammation, dermcidin is an integral component of the innate defense mechanism of human skin, functioning continuously rather than being triggered by external factors. Dermcidin is produced in the eccrine glands, released into sweat, and carried to the surface of the epidermis (38). Dermcidin levels are reduced in HS, possibly allowing for the overgrowth of certain bacteria (39).

S100 Proteins are a family of calcium-binding proteins involved in regulating various cellular processes, including cell growth and differentiation, protein phosphorylation, enzyme activities, and cytoskeleton dynamics (40). S100A4, S100A7, S100A8, S100A9, S100A12, and S100A15 are elevated in HS. Among these, S100A8 and S100A9 may serve as predictive biomarkers for adalimumab response in HS patients, while S100A15 could be a potential marker for disease severity and progression (41–45). Moreover, ribonuclease 7 (RNase 7) an epithelial-derived peptide with antimicrobial activity against Gram-positive bacteria, Gram-negative bacteria, and the yeast *Candida albicans* (46) is significantly elevated in HS patients (34, 47). Altered AMP production patterns, including elevated expression of the aforementioned S100A7, RNase7, and S100A8, along with an impaired response to microbial stimulants like muramyl dipeptide (MDP) and Pam2CSK4, result in elevated production of proinflammatory cytokines (IL-1β, IL-6, IL-8, TNF-α), contributing to continuous inflammation (47).

The complement system, a crucial part of the innate immune response, assists in tagging pathogens for destruction and promoting inflammation. The role of the complement system in HS pathogenesis is still under investigation, with conflicting evidence regarding local and systemic activation (48). Direct immunofluorescence (DIF) of HS lesions have found no significant local deposition of complement components C1q, C3c, C4d, and properdin. However, increased presence of C5aR1 positive neutrophilic granulocytes in HS lesions warrants further investigation (49).

## Keratinocytes

In addition, for being primary structural cells, keratinocytes also have profound immune functions (15, 45, 47, 50–53). Different layers of the skin in HS are associated with distinct inflammatory profiles, reflecting specific cellular compositions. In early lesions and/or at the surface of HS, keratinocytes in the epidermis act as key initiators of inflammation, by producing chemokines such as CCL3, CXCL3, and IL-8, which recruit immune cells, including neutrophils, CD8 T cells, and natural killer cells, to the epidermis. This localized inflammatory response establishes the early microenvironment that drives the progression of HS lesions (51).

A unique feature of HS is the presence of epithelialized tunnels present in the dermis that are characterized by a cylinder of keratinocytes with a central lumen. Tunnels are an active source of inflammation in HS that promote disease progression and drive the inflammatory response, leading to the production of chemokines and cytokines and immune cell infiltration (37). In HS dermal tunnel keratinocytes, it was shown that IL17C and IL1A were expressed at higher levels in the stratum corneum; IL6 was expressed at higher levels in the stratum spinosum, and IL-1β was expressed at higher levels in the stratum basale when compared to controls. Both HS epidermis and HS dermal tunnel keratinocytes expressed higher levels of IL1RL1 (ST2) compared to controls, with HS epidermis keratinocytes having even higher levels than HS dermal tunnel keratinocytes (54).

Keratinocyte hyperplasia in the outer root sheath (ORS) of hair follicles is a consistent feature in HS, often marked by increased expression of K19, indicative of hyperplastic keratinocytes in the infundibulum (55). Whether keratinocyte hyperplasia initiates inflammation or whether it is a consequence of inflammation has not been resolved. Additionally, abnormal keratinocyte responses, including epithelial-mesenchymal transition (EMT) and dermal presence of free keratinocytes, suggest involvement in tissue remodeling and wound healing processes. Inflammation drives keratinocyte proliferation across both interfollicular epidermis and follicular ORS, with follicular occlusion as a subsequent effect. Elevated matrix metalloproteinase levels (MMP-2, MMP-9) during the inflammatory response contribute to tissue degradation and remodeling. Dysregulation of keratinocyte function in HS is further underscored by reduced expression of keratin maturation markers (K2e, K10, K19) and adhesion molecules desmoglein 1(DG1) and desmocollin 1(DSC1) in inflamed epithelium, alongside decreased ICAM-1 levels and altered TGF-β receptor ratios on fibroblasts, thus impairing wound healing and promoting hypertrophic scarring (19).

The chemokines produced by keratinocytes recruit immune cells such as neutrophils, macrophages, and T cells to the lumen of tunnels. This influx of immune cells is particularly pronounced in more advanced stages of HS. Once immune cells are present in the epidermis, they produce a set of cytokines, such as IFN-γ and IL-17A, further perpetuating an inflammatory cycle (51). Treatment with Brodalumab, an IL-17RA antagonist leads to significant downregulation of epithelial proliferation, differentiation, and inflammatory cytokine production. This finding suggests that targeting IL-17 signaling effectively reduce the keratinocyte-mediated inflammatory loop in HS (56).

The chronic inflammation observed in HS lesions may be driven by the interaction between certain anaerobic bacteria, particularly *Fusobacterium nucleatum* (FN) and *Prevotella* species, and keratinocytes (57). This interaction results in the upregulation of several genes. Keratinocytes stimulated with FN show a distinct profile of upregulated genes, including IL-17 pathway-related genes (IL17C), pro-inflammatory cytokines (IL-6, TNF), and chemokines (CXCL1-8, CCL20). This profile is more pronounced and diverse compared to stimulation by Gram-positive bacteria, indicating a unique immune activation pattern specific to GNAs.

Studies in murine models and HS patient biopsies demonstrate that GNAs, particularly FN, induce a local inflammatory response resembling features observed in HS lesions. This response includes the recruitment of neutrophils and macrophages, as well as the production of IL-17 and other inflammatory mediators. Inhibition studies using TLR4 and JAK inhibitors show that blocking these pathways can attenuate the inflammatory response in keratinocytes stimulated by GNAs. These findings highlight potential therapeutic targets for mitigating HS-associated inflammation (57).

In summary, keratinocytes play an important role in the inflammatory response and overall pathophysiology of HS and should be considered in therapeutic targeting.

## Macrophages

The inflammatory response in HS involves significant infiltrates of macrophages and neutrophils to the affected area after follicular rupture (5, 58). Macrophages can be identified by CD68 positivity and are key producers of several pro-inflammatory cytokines including IL-1β, TNF-α, and IL-23 (59). In addition, upregulation of TLR2 on macrophages and dendritic cells in HS lesions has been observed. TLR2 activation leads to increased production of TNF-α and IL-12, contributing to the inflammatory cascade (60). Metalloproteinases (MMPs), particularly MMP-2 and MMP-9, are upregulated in HS. These enzymes, produced by macrophages and other cells, degrade extracellular matrix components, leading to tissue destruction and chronic wound formation (61). Differentially expressed genes associated with macrophage functions, such as phagocytosis and Fc receptor-mediated signaling, are significantly elevated in HS. HS lesions show a predominance of M1 macrophages, characterized by the upregulation of M1 markers like HLA-DRB5 and STAT1 and downregulation of M2 markers such as CD163 and MRC1 (62). Chronic inflammation and impaired wound healing in HS may be partly due to this transcriptional profile of macrophages. Genes related to phagocytosis and respiratory burst, such as Fc gamma receptor 1a (FCGR1A), Fc gamma receptor 1b (FCGR1B), formyl peptide receptor 1 (FPR1), and superoxide dismutase 2 (SOD2) are also upregulated in macrophages within HS lesions suggesting enhanced microbial killing and clearance of cellular debris. HS lesions exhibit upregulation of interferon-stimulated genes with both type I and II IFN activation sites, indicating that both IFN-α/β and IFN-γ pathways are active in HS macrophages. Genes involved in Fc receptor signaling, which are crucial for antibody-dependent cellular cytotoxicity (ADCC) and other immune responses, are also upregulated in HS macrophages (62). This upregulation suggests that Fc receptor-mediated signaling pathways may play a significant role in the heightened inflammatory state and chronicity of HS lesions. Dysregulation of these pathways contributes to persistent macrophage activation, impaired wound healing, and prolonged tissue damage (62). Therapeutically, targeting Fc receptor signaling or its downstream effects could represent a novel strategy to modulate immune responses and reduce inflammation in HS (6, 62). Furthermore, genes associated with Fc receptor signaling could serve as biomarkers for disease activity or treatment response (62). Overall, the inflammatory response in HS is driven by dysregulated macrophage activity, characterized by the overproduction of pro-inflammatory cytokines, upregulation of Fc receptor signaling pathways, and an imbalance between M1 and M2 macrophages, which collectively contribute to chronic inflammation, impaired wound healing, and tissue damage.

## Fibroblasts

The role of fibroblasts in HS is multifaceted and significant in the disease’s pathogenesis. Research on ligand-receptor interactions has revealed that fibroblasts in HS skin produce various chemokines, including CCL19, CCL20, CXCL2, and CXCL12. These chemokines bind to receptors on myeloid cells, indicating that fibroblasts play a significant role in attracting immune cells to the HS infiltrate (63).

Research analyzing the upregulation of canonical pathways and enriched Gene Ontology biological processes has identified two specific fibroblast subtypes, secreted frizzled-related protein 4 (SFRP4) and C-X-C motif chemokine ligand 13 (CXCL13) that significantly contribute to the immune response and fibrosis observed in HS (63). Notably, the SFRP4 fibroblast subtype is influenced by signaling from the Hippo pathway, which plays a profibrotic role in HS (63). Secreted frizzled related protein 2 (SFRP2) and C-X-C motif chemokine 12 (CXCL12+) fibroblast populations have been demonstrated to be expanded in HS lesional tissue compared to non-lesional tissue and healthy controls (64). These changes in fibroblast subpopulations are linked to the severity of HS and the presence of epithelialized tunnels. The expansion of SFRP2+ fibroblasts, which play a role in dermal homeostasis and inflammatory cell retention, may contribute to the formation of tertiary lymphoid structures (TLOs) and other histological features such as dermal layer thickening, fibrosis, and inflammatory cell accumulation. Increased CXCL12+ fibroblasts, associated with immune surveillance and Th2 immune responses, are found in severe disease stages and in tissues with epithelialized tunnels, indicating a role in heightened inflammation. SFRP1+ fibroblasts, which are important for extracellular matrix remodeling, are reduced in advanced HS, possibly due to the destruction of follicular units (64).

Despite these insights, several questions remain unanswered regarding the precise mechanisms by which these fibroblast subpopulations interact with other cell types within the HS microenvironment. Further research is needed to elucidate the functional roles of these fibroblast subtypes in chronic inflammation and fibrosis, as well as their potential as therapeutic targets in HS management.

## Neutrophils

Neutrophils play a crucial role in HS and are a primary source of IL-17 in HS lesions, despite their lower IL-17 expression levels compared to Th17 cells. The abundance of neutrophils results in substantial IL-17 release, which sustains and amplifies inflammation, creating a positive-feedback loop that enhances the production of additional proinflammatory molecules like S100A8 and S100A9 (15). While Th17 cells also produce IL-17, the deep infiltrates in HS lesions predominantly consist of IL-17+ neutrophils. Additionally, neutrophils contribute to inflammation through the formation of neutrophil extracellular traps (NETs), which are linked to increased immune dysregulation and inflammation (65–69).

Neutrophils show increased infiltration in the dermal tunnels of HS lesions compared to healthy controls. Immunohistochemical analysis reveals clusters of neutrophils surrounding the epithelialized tunnels, indicating active recruitment and migration towards the tunnel lumen. Activated neutrophils in tunnels are evidenced by strong staining for the neutrophil activation marker CD177. Furthermore, elevated levels of CXCL1 and CXCL8 throughout the tunnel epithelium suggest that these chemokines play a role in recruiting neutrophils into the tunnels (37).

Treatment with brodalumab, an IL-17RA antagonist, results in decreased expression in neutrophil-associated markers and pathways, including granulocyte chemotaxis and migration (56). Patients treated with brodalumab show a marked reduction in inflammatory cytokines like TNF and IL-8, indicating reduced systemic neutrophilic inflammation in HS. Higher baseline levels of LCN2, a neutrophil activity marker, in lesional skin correlate with a greater decrease in inflammatory cytokines after brodalumab treatment, suggesting LCN2 could be a biomarker for predicting response to IL-17RA blockade (56).

HS patients show a distinct serum proteomic profile compared to healthy controls, with elevated levels of neutrophil-related proteins like IL-17A, CXCL1, and Cathepsin D. Moreover, enrichment analysis revealed pathways significantly involved in neutrophil-mediated inflammation, such as neutrophil chemotaxis and degranulation. High serum levels of neutrophilic markers (LCN2, IL-17A) correlate with clinical severity scores in HS, suggesting their potential as biomarkers for disease activity. Elevated mRNA levels of CSF3 in HS lesional and perilesional skin correlate with increased neutrophil-related proteins in serum, indicating a skin-blood interaction driving neutrophilic inflammation (70).

Both perilesional and lesional HS skin exhibit significant neutrophilic infiltration, with upregulation of genes involved in neutrophil chemotaxis, migration, and extravasation. Furthermore, histological studies revealed no significant differences in leukocytic infiltration between perilesional and lesional skin, both of which have significantly higher infiltration than healthy control skin. Similarly, perilesional and lesional skin presented activation of the IL-17 pathway, with gene set variation analysis (GSVA) indicating IL-17 signaling even in non-lesional skin, and high LCN2 expression in lesional and perilesional skin identifies a more neutrophilic HS subtype, correlating with increased levels of neutrophil-related genes and pathways (71).

HS lesions exhibit increased counts of plasma cells, CD8+ T cells, neutrophils, and M0 and M1 macrophages compared to perilesional skin and healthy donor skin, indicating a robust inflammatory response. There is a significant correlation between the presence of plasma cells and neutrophils in HS lesions. The cytokine B-cell activating factor (BAFF) is highly expressed in HS lesions, with a strong link to neutrophil markers. Single-cell RNA-Seq data indicate that neutrophils are the primary source of BAFF in HS lesions. Neutrophils activated by G-CSF, particularly in the presence of bacterial components, produce high levels of BAFF, supporting the persistence and activation of plasma cells in HS lesions (72). The elevated expression of BAFF in HS lesions suggests its potential role in perpetuating the inflammatory environment by supporting plasma cell survival, migration, adhesion, and activation.

Recent studies have expanded the understanding of immune cell involvement in hidradenitis suppurativa, highlighting the role of mast cells in addition to other innate immune populations. Chu et al. identified mast cells as a predominant source of IL-17A in HS lesions, demonstrating their close interaction with IL-17 receptor-expressing keratinocytes and their role in promoting keratinocyte proliferation and disease-associated gene expression (73). Additionally, Kashyup et al. noted that mast cells contribute to the inflammatory microenvironment of HS, interacting with other immune cell populations within the IL-17 signaling pathway, further supporting their involvement in chronic inflammation (74). Flora et al. provided additional evidence by showing that mast cells are significantly upregulated in HS lesions, particularly in epithelialized tunnels and fibrotic regions, where they interact with neutrophils, B cells, and plasma cells to drive persistent inflammation (75). RNA sequencing and immunohistochemistry revealed a shift from resting to activated mast cells in HS lesions, with their numbers significantly reduced following treatment with the spleen tyrosine kinase (SYK) inhibitor Fostamatinib, suggesting a novel therapeutic approach (75). Together, these findings indicate that mast cells play a role in the inflammatory and fibrotic processes of HS and may represent a promising target for future treatment strategies.

## Molecular regulation of epigenetic mechanisms in HS

**Figure 4:**
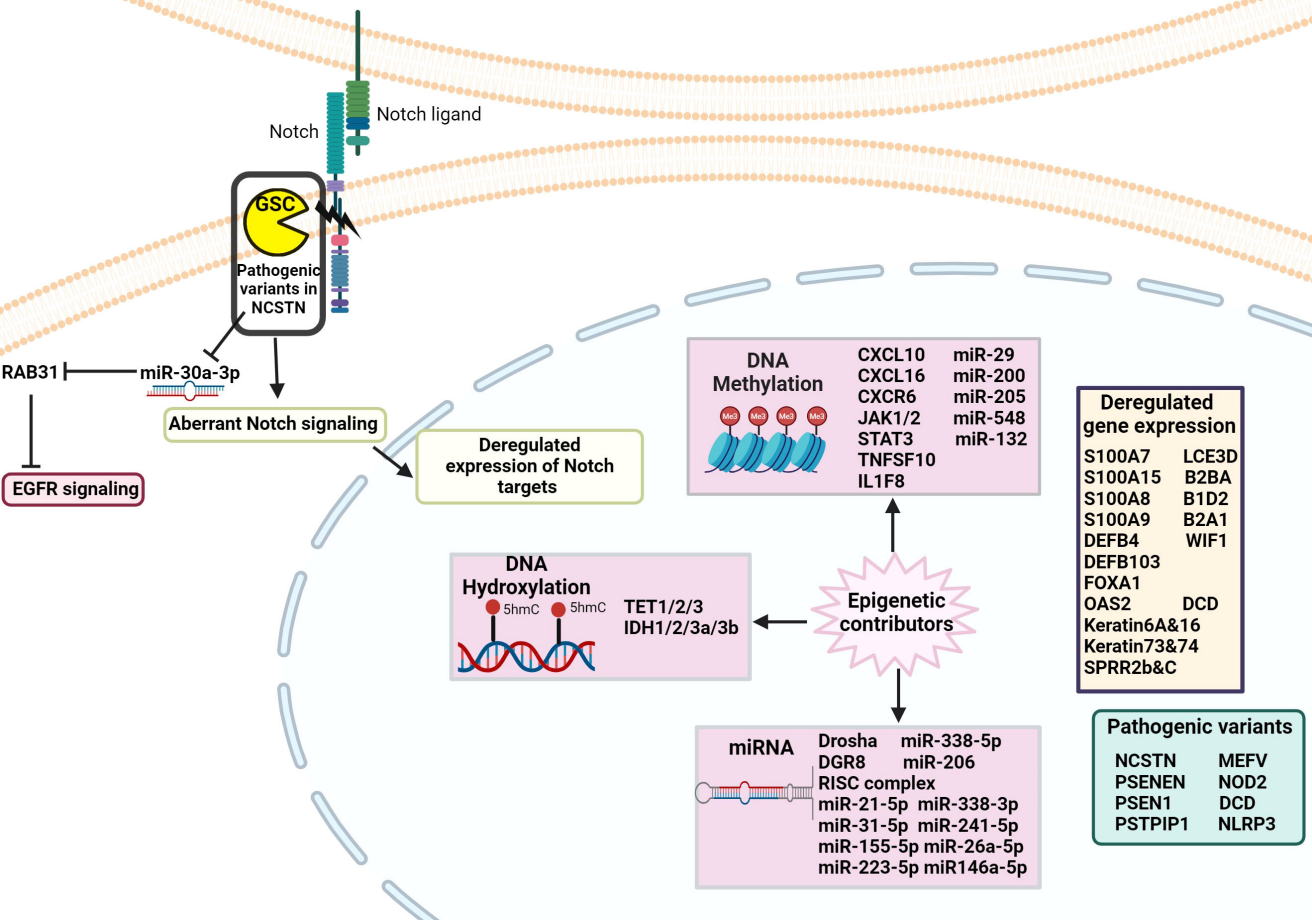
Schematic of the genetic and epigenetic alterations involved in the pathophysiology of HS. Pathogenic variants in genes, such as those in the NCSTN gene, have been identified in HS patients, leading to dysregulated Notch signaling. Epigenetic factors, including DNA methylation, hydroxylation, and disrupted miRNA expression result in abnormal regulation of gene expression that contributes to the development of HS pathology.

Epigenetics explores the inherited and acquired changes that affect gene activity without changing the DNA sequence. The human epigenome, which includes DNA methylation, histone modifications, and non-coding RNAs, regulates gene expression and is crucial for managing many cellular functions in health and disease states (50) (**Figure 4**).

DNA methylation regulates gene expression by recruiting proteins involved in gene repression or by inhibiting the binding of transcription factors to DNA. DNA methylation is essential for regulating tissue-specific gene expression, silencing retroviral elements, genomic imprinting, and other functions that contribute to cellular homeostasis. Studies have identified significant differential methylation patterns between HS patients and controls. These methylation sites are located in the promoter region, gene regulatory elements, and coding regions of various cytokine genes that encompass both pro-inflammatory and anti-inflammatory genes. Specific cytokine genes with differential methylation include chemokines (e.g., CXCL10), chemokine receptors (e.g., CXCR6), growth factors (e.g., JAK2), interferons (e.g., JAK1, STAT3), TNF family members (e.g., TNFSF10), and interleukins (e.g., IL1F8, IL1R2, IL17F, IL5RA) (76). A study conducted whole-genome DNA methylation sequencing on lesional and unaffected skin samples from HS patients. The study revealed that hypermethylation of the CXC chemokine ligand 16 (CXCL16) occurs. CXCL16 is a crucial mediator of innate immunity in epidermal keratinocytes and attracts CXC chemokine receptor (CXCR) 6-expressing cells, such as activated T cells and Natural Killer (NK) T cells (77). This hypermethylation led to decreased expression of CXCL16, potentially disrupting the chemotaxis of CXCR6-bearing cells and impairing innate and adaptive immune responses in HS patients. Furthermore, the downregulation of CXCL16, given its antimicrobial properties, could also facilitate bacterial colonization, thereby perpetuating the inflammatory cycle (78).

The role of telomere-related genes (TRGs) in the pathogenesis of HS has been explored, revealing significant disruptions in DNA methylation patterns that highlight their involvement in critical cellular processes. TRG’s are involved in DNA repair, telomere maintenance, mismatch repair, and cell cycle control. Researchers analyzed the methylomes of TRGs in genomic DNA derived from the whole blood of HS patients. They identified 585 differentially methylated sites in these genes, with 474 being hypomethylated and 111 hypermethylated (79). The disruption in gene function leads to telomere shortening, which is linked to the progression of HS, aging, cellular senescence, and an elevated risk of cancer.

Gene Ontology (GO) and KEGG pathway analyses based on epigenome-wide DNA methylation profiling of blood samples from 24 hidradenitis suppurativa (HS) patients and 24 age-, sex-, and ethnicity-matched controls, have identified several dysregulated pathways associated with HS, such as cytokine-cytokine receptor interaction, JAK-STAT signaling, MAPK signaling, chemokine signaling, TNF signaling, and IL-17 signaling (76). The dysregulation in cytokine gene methylation may explain various HS-associated comorbidities, including non-alcoholic fatty liver disease (NAFLD), depression, anxiety disorders, and wound healing impairment. In addition, adalimumab, the only TNF-α antagonist approved by the FDA and the European Medicines Agency (EMA) for moderate-to-severe HS, highlights mTORC1’s involvement in HS pathogenesis and its potential role on the efficacy of TNF-α inhibition (80).

Recent research has also explored the role of DNA hydroxylation in HS, shedding light on its potential impact on gene expression and disease progression. It has been reported that mRNA levels of Tet1/2/3 and IDH1/2/3a/3b are notably reduced in HS lesional areas compared to normal skin. Additionally, IDH1 and IDH2 expression was diminished in HS perilesional skin, and Tet3 levels were significantly lower in HS lesional regions in contrast to HS perilesional areas (81). Another study found that 5-hydroxymethylcytosine 5-(hmC) levels were significantly lower in both lesional and perilesional HS skin compared to healthy controls, with no significant difference between the lesional and perilesional HS skin. These findings suggest that DNA hydroxymethylation imbalances may contribute to the pathogenesis of HS, highlighting the need for further research on this mechanism and its regulatory enzymes to better understand the inflammatory processes in HS (82).

Histone acetylation has emerged as another key epigenetic mechanism implicated in HS pathogenesis. Dysregulated histone acetylation patterns have been linked to persistent inflammation, defective wound healing, and immune dysregulation (83). For instance, abnormal acetylation in genes associated with cytokines and chemokines, such as IL-1β and TNF-α, contributes to the regulation of inflammatory responses. Moreover, aberrant acetylation of genes involved in extracellular matrix remodeling, angiogenesis, and keratinocyte proliferation can impair the resolution of lesions and perpetuate tissue damage (83). For example, inhibition of histone deacetylases (HDACs) has shown potential in modulating inflammatory responses in HS and other inflammatory skin disorders (84). Targeting these mechanisms with precision epigenetic therapies, such as HDAC inhibitors could offer promising avenues for intervention in HS. In addition to histone modifications and DNA methylation, recent studies highlight the role of long non-coding RNAs (lncRNAs) in the epigenetic regulation of HS (85). The differential methylation of lncRNAs in patients with HS suggests that these molecules may critically influence inflammation, keratinocyte differentiation, and immune responses, thereby contributing to HS pathogenesis (86).

## MicroRNAs

In the skin of HS patients, miRNAs present significant decrease in the expression of key miRNA maturation regulators. Drosha is a nuclear RNase III enzyme required for the maturation of miRNAs (87). Studies have demonstrated that Drosha is downregulated in HS lesional skin. In addition, DGRC8, a cofactor for Drosha in primary miRNA processing, shows decreased expression in seemingly healthy perilesional skin of HS patients (88). This indicated their potential role in an underlying subclinical inflammatory response.

RNA-induced silencing complex (RISC) process pre-microRNAs into mature miRNAs. A study examined the expression levels of RISC components, including trans-activation response (TAR) RNA binding protein 1 (TRBP1), TRBP2, protein Activator of the interferon-induced protein kinase (PACT), Argonaute RISC Catalytic Component-1 (AGO1), Component-2 (AGO2), metadherin, and staphylococcal nuclease and Tudor domain-containing-1 (SND1). The results revealed a significant reduction of these components in HS lesional skin compared to healthy controls, indicating the involvement of miRNAs in the pathogenesis of HS (89).

Notable differences between HS patients and healthy controls have been identified in 60 CpG sites corresponding to 65 unique microRNA genes. Among these differences, 54 CpG sites were hypomethylated, and 6 were hypermethylated. Some of the critical microRNAs identified for skin function included miR-29, miR-200, miR-205, miR-548, and miR-132. The miR-200c gene plays an essential role in regulating skin repair after injury and may influence age-related changes in wound healing and miR-132 shows significant upregulation during the inflammatory phase of wound healing, boosting the activity of the STAT3 and ERK pathways, which are crucial for keratinocyte proliferation (90).

Furthermore, differential expression patterns of specific miRNAs have been observed in HS patients. One study found overexpression of miRNA-21-5p, miRNA-31-5p, miRNA-155-5p, and miRNA-223-5p, in HS patients compared to healthy controls (91). Another study identified miR-338-5p overexpression in HS, correlating with increased expression of IL-1a, IL-6, and COX2 (92). In contrast, miR-206, miR-338-3p, miR-24-1-5p, and miR-26a-5p are reduced in peripheral blood leukocytes from HS patients (92). Studies have also found decreased expression of miRNA-146a-5p in peripheral blood leukocytes and increased expression of miRNA-146a-5p in HS lesional skin (91, 92). The study by Liang et al. utilized gene expression profiles from control and HS skin samples, employing machine learning to pinpoint molecular factors contributing to HS pathophysiology (93). Key findings were the identification of KYNU gene and a regulatory pathway involving miR-382-5p, KYNU and MUC19 genes (93). KYNU, a critical enzyme in the kynurenine pathway of tryptophan metabolism, was found to play a pivotal role in the pathogenesis of HS by linking metabolic processes to inflammation and immune dysregulation (93). This process is further modulated by miR-382-5p, a microRNA that interacts with KYNU and potentially regulates its expression, where dysregulation may exacerbate inflammatory pathways in HS (93). Additionally, MUC19, a mucin-encoding gene, forms part of this RNA network, adding complexity to the molecular mechanisms underlying HS and shedding light on the epithelial barrier dysfunction associated with the disease (93).

The dysbiotic microbiome in HS may contribute to miRNA deregulation by influencing key pathways involved in immune responses and inflammation. For example, increased abundance of microbial species capable of activating TLR2 and TLR4 pathways could lead to altered miRNA profiles, as these pathways are known to regulate immune-related miRNAs (60, 94). Additionally, dysbiotic genera like *Prevotella* and *Porphyromonas* have been shown to induce cytokine production such as IL-1β and IL-6, which in turn can modulate miRNA expression patterns (95–98). This dysbiotic-induced miRNA dysregulation is supported by findings showing differential miRNA expression patterns in HS lesional and perilesional skin (88, 91). Dysbiosis may amplify chronic inflammation and subclinical inflammatory responses through its influence on miRNAs, creating a feedback loop that perpetuates disease pathology.

## Molecular regulation of genetic mechanisms in HS

HS was initially thought to be a Mendelian autosomal inherited disorder that was largely based on familial aggregation and genetic analysis in multiple families (99, 100). The HS locus was first mapped to chromosome 1p21.1–1q25.3 in a Chinese four-generation family (101). Nicastrin (*NCSTN*) was identified as a specific gene at 1q23.2, along with *PSENEN* (Presenilin Enhancer, 19q13.12) and *PSEN1* (Presenilin 1, 14q24.2) (102). Additional pathologic variants of *NCSTN*, *PSEN1*, and *PSENEN* have been identified in familial HS patients (102–106). In addition, Gamma-secretase complex (GSC) mutations that affect Notch signaling pathways crucial for epidermal homeostasis and differentiation are implicated in HS pathogenesis. Pathogenic variants in *NCSTN*, that codes for a subunit of GSC, result in reduced miR-30a-3p levels, which subsequently increasing the expression of Ras-Related Protein Rab-31 (RAB31). Elevated levels of RAB31 were found to hasten the degradation of activated epidermal growth factor receptor (EGFR), causing abnormal keratinocyte differentiation. From these observations, the authors inferred that the NCSTN/miRNA-30a-3p/RAB31 axis in familial cases of HS likely disrupts the EGFR signaling pathway, leading to irregular keratinocyte differentiation (107). Moreover, data from a single family with *NCSTN* polymorphisms show Notch and PI3K/AKT downregulation linked to inflammation and keratinocyte hyperplasia (105). Genetic studies have also identified pathogenic variants in *MEFV* and *NOD2* genes in familial HS, suggesting a polygenic presentation (108). Sporadic HS cases have a significant genetic background involving immune response genes like *TNF* and *TLR4*, contributing to disease susceptibility (109). Recent polygenic score analyses reveal that common variants, particularly those enriched in cell adhesion-related genes, also contribute to the genetic architecture of sporadic HS. (110). Recent genome-wide association studies (GWAS) have further expanded our understanding of the genetic architecture of HS, identifying genetic loci near the *SOX9* and *KLF5* genes, implicating their roles in epidermal differentiation and follicular inflammation in HS pathogenesis (111). Additionally, the study confirmed a strong heritability component in HS, with siblings of affected individuals having a nearly 20-fold increased risk of developing the disease. While γ-secretase complex mutations account for less than 5% of cases, common genetic variants near these loci may play a larger role in disease susceptibility (111). Syndromic HS forms, including Pyoderma gangrenosum, Acne, and Suppurative Hidradenitis (PASH) and Pyogenic Arthritis, Pyoderma gangrenosum, Acne, and Suppurative Hidradenitis (PAPASH) syndromes, involve genetic changes in *PSTPIP1*, *NLRP3*, and other autoinflammatory genes (112, 113). Further supporting the role of autoinflammation in HS, Vural et al. identified a significantly increased prevalence of *MEFV* gene mutations in patients with complex HS, particularly those with severe (Hurley stage III) disease or additional inflammatory symptoms (114). These findings suggest that mutations in *MEFV*, a gene associated with Familial Mediterranean Fever, may contribute to dysregulated inflammasome activation in HS, reinforcing the hypothesis that autoinflammatory mechanisms underlie certain HS phenotypes. While these genetic findings highlight the role of hereditary and autoinflammatory mechanisms in HS, the extent to which genetics alone determines disease progression remains unclear. Factors such as BMI and smoking have been more strongly associated with disease progression than familial HS status, suggesting that environmental influences significantly modify genetic susceptibility in HS (115). This reinforces the need to consider both genetic and non-genetic risk factors when evaluating disease trajectory.

HS associated with other diseases like Dowling–Degos disease (DDD), implicates genes such as *POFUT1* (Protein O-Fucosyltransferase 1), *POGLUT1* (Protein O-Glucosyltransferase 1), and *PSENEN*. These genes may increase disease susceptibility by influencing Notch signaling (116, 117). A pilot study investigating epigenetic age in HS found that immune-related changes in the skin, rather than non-immune aging pathways, may accelerate epigenetic aging in HS skin compared to control skin, and that increased PhenoAge Acceleration in HS skin could serve as a biomarker for current and future morbidity in HS patients (118, 119).

Several other genes have been shown to exhibit significant changes in expression. *S100A7* is significantly elevated, and integrative transcriptome analyses of HS lesions have demonstrated that *S100A15* is also a significantly upregulated gene found in lesional HS skin (39, 43). Additionally, S100A8 and S100A9 proteins involved in antimicrobial responses, psoriasis, and wound healing, are overexpressed in HS (41). Moreover, β-defensin genes, including *DEFB4* and *DEFB103*, which encode for the proinflammatory mediators human β-defensin–2 and human β-defensin–3 respectively, are strongly upregulated among HS patients (39, 120). Furthermore, Oligoadenylate synthetase 2 (*OAS2*), an interferon-stimulated gene with antiviral properties, has been identified as being upregulated in HS lesional skin relative to HS non-lesional skin (39). In contrast, *DCD* is one of the most significantly downregulated genes in HS (39).

## Conclusion

HS involves a multifactorial interplay between immune responses, cellular dysfunction, and genetic predispositions. Recent advancements in our understanding of HS have highlighted the significant role of epigenetic mechanisms, particularly DNA methylation, histone modifications, and non-coding RNAs, in regulating the inflammatory responses characteristic of this disease. These epigenetic changes contribute to the persistent activation of immune cells and the aberrant expression of cytokines and chemokines that drive the chronic inflammatory state observed in HS lesions.

Inflammasomes, particularly the NLRP3 inflammasome, are critical mediators of the inflammatory process in HS. The dysregulation of inflammasome activity not only promotes the release of pro-inflammatory cytokines but also perpetuates the inflammatory milieu, contributing to the formation of the characteristic abscesses and tunnels in HS. Thus, targeting inflammasome pathways for the treatment of HS has emerged as a potential therapeutic strategy, offering new avenues for mitigating the inflammatory burden in HS patients.

Epigenetic memory refers to the heritable alterations in gene expression that do not involve changes to the underlying DNA sequence, driven by mechanisms such as DNA methylation, histone modification, and non-coding RNA activity. These changes are influenced by environmental and metabolic factors, which leave lasting marks on gene regulatory networks. Emerging evidence highlights the relevance of epigenetic memory in the context of inflammatory skin diseases and its potential impact on recurrent and chronic conditions like HS (121–124). In inflammatory skin diseases, epigenetic memory plays a critical role in shaping the behavior of epidermal stem cells and their response to environmental insults. For instance, prior exposure to inflammatory stimuli can prime keratinocytes and immune cells, altering their reactivity to subsequent insults. This priming is often mediated by persistent histone modifications and changes in chromatin accessibility, allowing rapid transcriptional activation of inflammatory pathways upon re-exposure (124). Such mechanisms can contribute to defective barrier repair and chronic inflammation in conditions like HS, where impaired epidermal stem cell function perpetuates disease activity.

Factors such as smoking, obesity, and diabetes are known to influence epigenetic landscapes and may exacerbate chronic inflammatory conditions, including HS, by reinforcing epigenetic memory of inflammatory and metabolic dysfunctions. Smoking induces persistent epigenetic changes, primarily via alterations in DNA methylation. For instance, genes such as *AHRR*, *F2RL3*, and *GPR15*, which are associated with immune responses and metabolic regulation, exhibit altered methylation patterns correlated with smoking history (125). These epigenetic modifications are linked to heightened levels of pro-inflammatory cytokines like IL-2 and IL-13, which may exacerbate systemic and localized inflammation (125). Moreover, the effects of smoking on DNA methylation and cytokine profiles persist even after cessation, creating a durable epigenetic memory that could influence chronic disease progression (125).

Type 2 diabetes mellitus (T2DM) alters the epigenetic profile of dermal fibroblasts, impairing their ability to respond to inflammatory stimuli and repair wounds. Diabetic fibroblasts retain epigenetic marks that drive pro-inflammatory cytokine production and reduce their responsiveness to anti-inflammatory signals. This phenomenon, referred to as “epigenetic metabolic memory,” impacts barrier repair processes and likely contributes to the poor healing outcomes observed in diabetic ulcers (126). For example, T2DM fibroblasts show increased basal expression of pro-inflammatory cytokines and reduced sensitivity to inflammatory stimuli like TNF-α, which exacerbates chronic inflammation and impairs wound healing (126). Such alterations may also influence HS pathogenesis, as impaired wound healing and chronic inflammation are hallmarks of the condition.

Obesity drives systemic inflammation and metabolic dysfunction through epigenetic modifications, including altered methylation of genes like *PPARγ* and *GLUT4* and changes in histone acetylation that regulate metabolic pathways and inflammatory responses (127). Obesity-induced epigenetic memory reprograms adipose-resident macrophages to adopt a persistent pro-inflammatory state, exacerbating systemic inflammation even after weight loss (128, 129). This phenomenon, characterized by the retention of obesogenic memory, is particularly relevant to HS, as obesity-driven epigenetic changes may amplify disease severity and recurrence. Studies suggest that free fatty acids, such as stearic acid, induce metabolic reprogramming and a pro-inflammatory state through Toll-like receptor 4, further reinforcing these epigenetic modifications (129).

Investigating epigenetic memory in HS could provide valuable insights into the mechanisms underlying its recurrent nature and identify novel therapeutic targets. These targets may focus on modulating epigenetic memory to achieve sustained remission. Moreover, the investigation of how epigenetic memory influences inflammasome activation and other immune pathways in HS holds promise for uncovering new strategies to disrupt the vicious cycle of chronic inflammation in this debilitating condition.
